# The ulnar collateral ligament response to valgus stress, repetitive pitching, and elbow muscle contraction in asymptomatic baseball pitchers

**DOI:** 10.1016/j.xrrt.2023.11.005

**Published:** 2023-12-16

**Authors:** Bart van Trigt, Jeffrey van Goethem, Michel (M.P.J.) van den Bekerom, DirkJan (H.E.J.) Veeger, Marco (M.J.M.) Hoozemans

**Affiliations:** a Department of biomechanical Engineering, Delft University of Technology, Delft, the Netherlands; b Department of Human Movement Sciences, Faculty of Behavioural and Movement Sciences, Vrije Universiteit Amsterdam, Amsterdam Movement Sciences, Amsterdam, the Netherlands; c Department of orthopedic surgery, OLVG, Amsterdam, the Netherlands

**Keywords:** Baseball, Elbow, Injury mechanism, Tommy John surgery, Ultrasonography, Humeroulnar joint gap, Handgrip force

## Abstract

**Background:**

In baseball, repetitive pitching leads to medial elbow injuries, particularly to the ulnar collateral ligament (UCL). To prevent pitchers from UCL injuries, it is important to quantify the response to elbow stress. Repetitive elbow external valgus torque and muscular fatigue induced by repetitive pitching could affect markers of the response, that is, humeroulnar joint gap and UCL morphology. The aims of the study were three-folded: to investigate the effect of (1) exerted handgrip force on the humeroulnar joint gap, (2) repetitive pitching on the humeroulnar joint gap and the UCL morphology, and (3) exerted handgrip force on the humeroulnar joint gap for different levels of elbow valgus stress is different after compared to before repetitive pitching in asymptomatic baseball pitchers.

**Methods:**

Medial elbow ultrasound images were collected in 15 asymptomatic male baseball pitchers. Three levels of static elbow valgus stress (0N, 50N, 100N) were applied with a TELOS device before and after repetitive pitching and with or without handgrip force. These images were used to assess the humeroulnar joint gap size and UCL length and thickness. After 110 fastball pitches or when 80% self-perceived fatigue on a VAS scale was reached, participants were instructed to stop throwing. Repeated measures ANOVAs were used to statistically test significant differences.

**Results:**

Handgrip force did not significantly affect the humeroulnar joint gap. The UCL thickness and length and the humeroulnar joint gap were also not different after compared to before repetitive pitching. While higher levels of applied valgus stress significantly increased the humeroulnar joint gap (*P* < .001), this effect was not significantly different in the interaction with handgrip force and repetitive pitching.

**Conclusion:**

The humeroulnar joint gap changes for different levels of elbow valgus stress. However, adult baseball pitchers did not respond to elbow stress after a single pitching session with or without submaximal handgrip force in the humeroulnar joint gap and UCL morphology.

In baseball, pitchers may experience discomfort or pain during the baseball season while continuing to play. This frequently results in injuries to the musculoskeletal system. In Major League Baseball pitchers, around 28% of player disabilities were due to elbow injuries^6^ resulting in losses of 1.9 to 3.9 million dollars per player.^17^ The most common surgery in the treatment of elbow injuries is the Tommy John surgery, which is performed to recover the function of the insufficient ulnar collateral ligament (UCL). In professional baseball, 25% of the Major League Baseball pitchers and 15% of the Minor League Baseball pitchers have a history of such a surgery.^7^

It is not surprising that pitching in baseball is associated with a high incidence of elbow injuries and surgeries. Pitching exerts great forces on the human body and in particular on the medial structures of the elbow.^2^ In the late cocking or early acceleration phase of the pitch, the shoulder is positioned at maximal external rotation. In combination with accelerations, angular velocities, and inertia in the performance of the pitch, this results in an external elbow valgus torque.^8^ During pitching, peak external valgus torques around 50Nm are reported.^1^^,^^24^ This peak torque stresses the medial side of the elbow and produces a compressive force on the lateral side. This external valgus torque is resisted by the UCL as a structural stabilizer and the forearm flexor-pronator muscles (flexor carpi ulnaris, flexor digitorum superficialis, flexor carpi radialis, and pronator teres) as functional stabilizers.^26^ The interaction between the structural and functional stabilizers and the joint geometry counteracts the external valgus torque.^26^ Thus, in the evaluation of the medial elbow load, that is, the external valgus torque, in baseball pitching it is, in addition to the frequently treated UCL, important to consider the influence of elbow muscles.

Hattori et al (2020) investigated the effect of the forearm flexor pronator muscles on the humeroulnar joint gap by exerting handgrip force while measuring the joint gap. While participants were in a supine position and only gravity induced valgus stress on the medial side of the elbow, exerting maximal handgrip force decreased the humeroulnar joint gap.^11^ This indicated a stabilizing effect of the forearm flexor-pronator muscles on the elbow joint in a static position. During pitching, the flexor-pronator muscles show activity of 20-40% of their maximal voluntary contraction.^22^^,^^25^ although muscle mechanics are different in a dynamic movement, it is interesting to investigate the effect of submaximal handgrip forces in a static position as this might better reflect the flexor-pronator muscles force during pitching. In addition, the elbow is exposed to much higher elbow valgus torques while pitching compared to those caused by gravity in the experiment of Hattori et al (2020). However, it is unknown whether the elbow muscles, while being active because of gripping, can counteract the valgus torque under higher levels of valgus stress, and thus shield the UCL from high stresses.

Ultrasound imaging is used to investigate the humeroulnar joint gap and UCL morphology.^4^^,^^12^ When sustaining an overuse UCL injury, ultrasound images can show a change in morphology of the UCL, with complete tears of the UCL showing a ring-down artifact.^14^ In addition, pitchers with a UCL tear show a greater humeroulnar joint gap with a manually applied static valgus stress in comparison to the humeroulnar joint gap of asymptomatic pitchers.^21^ Ultrasound imaging, a noninvasive method, can thus be helpful in the study of the effects of repetitive pitching on structures of the medial elbow. Although it is yet not possible to measure the UCL morphology or the humeroulnar joint gap during baseball pitching, it is possible to measure the responses to elbow stress of repetitive pitching using static ultrasound imaging.^4^^,^^5^^,^^18^ During seasonal load, the UCL responds to stress by becoming thicker and the humeroulnar joint gap increases, on the contrary, during off-season rest, the UCL becomes thinner and the humeroulnar joint gap decreases.^4^ This shows that the UCL morphology, that is, UCL thickness, adapts to seasonal changes in exposure to elbow stress. Whether changes in UCL morphology can also be observed directly after a single training session with repetitive pitching is unclear.

The humeroulnar joint gap in youth baseball pitchers significantly increased when pitching 60 balls, which became even more clearly visible after 100 pitches.^10^ Although the authors concluded that this was likely due to muscle fatigue, they did not investigate the effect of handgrip force on the humeroulnar joint gap before and after repetitive pitching as an indicator of elbow muscle fatigue. Investigating the effect of elbow muscle activity, by exerting handgrip force, on the humeroulnar joint gap before and after a repetitive pitching session could quantify the fatiguing effect of such a session on elbow muscles and the effectiveness of their potential shielding effect with respect to repetitive UCL loading. If at the same time, the fatigued elbow muscles are less capable of counteracting elbow valgus torque during pitching, this might result in within-session changes in UCL morphology, which together with the humeroulnar joint gap might be assessed using ultrasound imaging directly before and after a repetitive pitching session.

Therefore, the aims of this study were, first, to investigate the effect of the exertion of handgrip force on the humeroulnar joint gap for different levels of elbow valgus stress in asymptomatic baseball pitchers before pitching. Second, we aimed to investigate whether repetitive pitching affects UCL thickness and length and the humeroulnar joint gap for different levels of elbow valgus stress in asymptomatic baseball pitchers. The third and final aim of the present study was to investigate whether the effect of exerted handgrip force on the humeroulnar joint gap for different levels of elbow valgus is different after compared to before repetitive pitching in asymptomatic baseball pitchers.

## Methods

### Participants

Fifteen asymptomatic male baseball pitchers participated in this study. Their mean age was 24.5 years (standard deviation [SD] 7.5, range 17-44), body height 191 cm (SD 5, range 183-199), and body mass 79.4 kg (SD 9.2, range 62.7-102.5). Most participants were pitching at a recreational level, with two participants playing at the highest level in the Netherlands. They played baseball for an average of 15.5 years (SD 7.6) and had pitching experience of an average over 11.5 years (SD 8.4). None of the participants had experienced any musculoskeletal injuries in the past six months nor received elbow surgery in the past. There were 11 right-handed pitchers and 4 left-handed pitchers. Participants signed an informed consent form before the data were collected. Ethical approval was granted by the Human Research Ethics Committee of the TU Delft on June 6, 2021.

### Procedure

A controlled laboratory study was performed in which all participants underwent the same procedure. Three different levels of static elbow valgus stress, with and without handgrip force using a hand-held dynamometer, were applied using a TELOS device (Telos GA-IIE stress device; Telos, Weiterstadt, Germany). To investigate the humeroulnar joint gap and the UCL morphology, ultrasound imaging was used before and after pitching a minimum of 60 fastballs.

**Figure 1:**
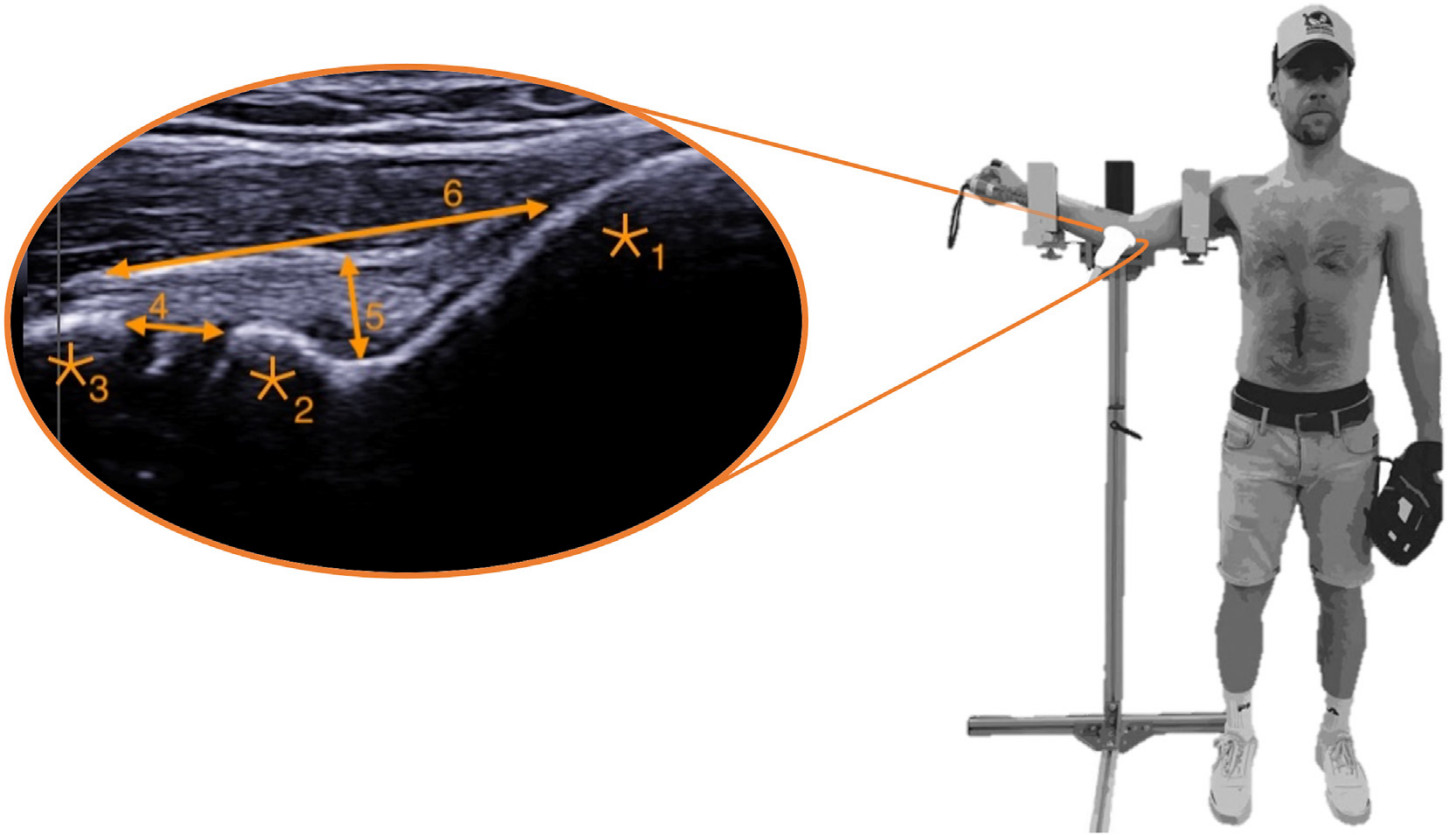
The right side of this figure shows the setup of the TELOS device on a self-build stand. The right-handed participant stands with the dynamometer in his hand. The ultrasound probe is positioned on the medial side of the elbow. The left figure shows the ultrasound image of the medial elbow, with 1. Medial epicondyle; 2. Humeral trochlea; 3. Sublime tubercle; 4. Humeroulnar joint gap; 5. UCL thickness; 6. UCL length. *UCL*, ulnar collateral ligament.

Before the first ultrasound measurement, the TELOS device was adjusted to the anthropometric characteristics of the participant (Fig. 1). The device was adjusted to the participant’s body height while standing, with the upper arm at 90 degrees abduction, the elbow flexed at 30 degrees, and the forearm supinated to imitate the pitching posture near the posture of maximal external rotation at which the highest valgus stress levels are expected during pitching (Fig. 1). To optimize the standardization of the ultrasound measurement with the participant in the TELOS device before and after a series of repetitive pitching, the orientation and position of the ultrasound probe were marked with a Sandel marker (Petite skin marker; Ansell, Iselin, NJ, USA) on the skin of the participant's arm.

While being fixated in the TELOS device, the device was subsequently set at three elbow valgus stress conditions: 0 N, 50N, and 100N. For each of the valgus stress levels, ultrasound images of the medial part of the elbow were taken. Then, to measure the condition ‘with grip force,’ participants were instructed to squeeze a hand-held dynamometer (JAMAR; Patterson Medical, Warrenville, IL, United States) to their maximum ability while being fixated in the TELOS device. From this reading, 20% was determined to be a pain-free and comfortable level. Again, with the participant in the TELOS device and exerting a handgrip force at 20% of their maximum effort, ultrasound images were collected at the imposed valgus stress levels of 0N, 50N, and 100N. After this first sequence of collecting ultrasound images, the participant started his warm-up.

In the next part of the experiment, participants had to perform fastball pitches from a pitching mound toward a fictional strike zone (71∗43 cm) at 18.66 meters. Ball speed was measured behind the strike zone using a radar gun (ACMI002; Applied Concepts Inc., Plano, TX, USA). The pitches were carried out in a series of 10 pitches. Before the start and between these 10 pitches, participants were asked to indicate their level of self-perceived fatigue using a visual analog scale. The minimum required number of pitches for the experiment was 60 fastball pitches. Participants were instructed to stop pitching when throwing more than 110 fastballs or when their visual analog scale score reached 80%. After the final pitch, the same sequence of collecting ultrasound images as before pitching was performed.

### Data collection

A Samsung ultrasound machine (HM70A; Samsung, Seoul, Republic of Korea) with a 12.3 MHz probe (LA3-16AD; Samsung, Seoul, Republic of Korea) was used to collect images of the medial side of the elbow. The ultrasound settings were set at 2.5 cm depth with a frequency of 12.3 MHz for optimal images (Fig. 1). A total of 36 ultrasound images for each participant were collected, 18 before and 18 after pitching. The 18 images included the three conditions of elbow valgus stress (0N, 50N, 100N), with and without exerted handgrip force, and three images per condition. All ultrasound images were taken by one examinator (J.v.G.), considering that the interobserver reliability was found to be sufficient for taking images of the anterior UCL with ultrasound.^9^ After each ultrasound image, the examinator completely removed the ultrasound probe from the participant’s elbow and repositioned the probe for the next image. Another investigator controlled the TELOS device to ensure proper elbow positioning for each condition.

### Data analysis

All ultrasound images of the medial side of the elbow were analyzed separately by two investigators (B.v.T. and J.v.G.). To prevent the researchers from confirmation bias on the different conditions, a randomizer script in Matlab was used to randomize all ultrasound images (Matlab 2019; Mathworks, Inc., Natick, MA, USA).

Fig. 1 shows an ultrasound image of the medial side of the elbow. Bony landmarks, such as the medial epicondyle, the humeral trochlea, and the sublime tubercle, were determined before drawing lines to determine the humeroulnar joint gap, the UCL thickness, and the UCL length. ImageJ (ImageJ; U.S. National Institutes of Health, Bethesda, MD, USA) was used to determine the UCL thickness, UCL length, and humeroulnar joint gap width in mm. This software enables the researchers to calibrate each image to set a pixel/mm ratio (16.2 pixels/mm). After analysis, the key for randomization of the images was shared between the two researchers and applied to the dataset. From the 36 ultrasound images, each of the conditions had three measurements from which the mean was calculated. This resulted in 12 data points. The intraclass correlation coefficient for absolute agreement using a random effects model was determined for the three outcome variables to determine the inter-rater reliability of the data analysis (Table I). The results can be considered acceptable.^19^

**Table I:** Intraclass correlation coefficients between the two researchers for the three outcome variables.

	ICC	CI
Humeroulnar joint gap	0.75	0.68-0.82
UCL length	0.81	0.66-0.89
UCL thickness	0.82	0.65-0.89

*CI*, confidence interval at 95%; *ICC*, intraclass correlation coefficient; *UCL*, ulnar collateral ligament.

### Statistical analysis

The dataset of the investigator J.v.G. was used for the statistical analysis, considering the sufficient level of reliability for the data analysis. To analyze the effect of handgrip force on the humeroulnar joint gap for the different levels of elbow valgus stress before pitching, a two-way (handgrip force [without, with] x elbow valgus stress [0N, 50N, 100N]) repeated measures analysis of variance (ANOVA) was used. To study the effect of repetitive pitching on UCL morphology and the humeroulnar joint gap for different levels of elbow valgus stress, a two-way (time [before pitching, after pitching] x elbow valgus stress [0N, 50N, 100N]) repeated measures ANOVA was used. To examine whether the effect of exerted handgrip force on the humeroulnar joint gap for the different levels of elbow valgus was different after compared to before repetitive pitching, a three-way (handgrip force [without, with] x elbow valgus stress [0N, 50N, 100N] x time [before pitching, after pitching]) repeated measures ANOVA was applied. Bonferroni post hoc pairwise comparisons were conducted to examine the significant interaction. The sphericity assumptions were valid and the data were normally distributed according to the Shapiro-Wilks tests and visual inspections of the histograms, q-q plots, and box plots. Significant differences were set at a level of *P* < .05. Data were statistically analyzed using Jamovi (version 2.3; Jamovi Project, Sydney, Australia) and visualized with R Software (version 2022.2.0.443; R Studio, Boston, MA, USA).

## Results

Data regarding the humeroulnar joint gap from a total of thirteen of the fifteen participants were included in the statistical analysis (n = 13). The researchers reached a consensus that the data of two participants were not sufficient in terms of the quality of the images to accurately measure the humeroulnar joint gap. The data regarding UCL thickness and UCL length were obtained and included in the statistical analysis for all participants (n = 15). A total of 1260 fastballs were pitched, ranging from 60 to 110 fastballs per participant. The average ball speed was 108.0 kph (SD 7.1) (67.1 mph, SD 4.4).

### Effect of handgrip force on the humeroulnar joint gap

Fig. 3*A* shows the mean humeroulnar joint gap in mm of 13 participants for 0N, 50N, and 100N of imposed elbow valgus stress using the TELOS device, with and without exerted handgrip force. The mean humeroulnar joint gap varies from 3.01 (SD .77) mm to 4.24 (SD 1.11) mm. There was a significant main effect of elbow valgus stress, showing that the humeroulnar joint gap increases with increasing levels of elbow valgus stress (Table II). Although the mean humeroulnar joint gap decreased with handgrip force at all levels of elbow valgus stress, the main effect of handgrip force and the interaction between handgrip force and elbow valgus stress were not significant for the humeroulnar joint gap (Table II).

**Table II:** Main and interaction effects for the repeated measures ANOVA concerning the effects of elbow valgus stress and handgrip force on the humeroulnar joint gap before repetitive pitching.

	F-value (df)	*P* value	Effect size (η^2^)	Bonferroni		
				0N–50 N	0N–100N	50–100N
Elbow valgus stress	21.14 (2,24)	*P* < .001	0.638	*P* = .001	*P* < .001	*P* = .123
Handgrip force	0.45 (1,12)	*P* = .515	0.036			
Elbow valgus stress∗ Handgrip force	0.31 (2,24)	*P* = .738	0.025			

*ANOVA*, analysis of variance.

### UCL morphology and humeroulnar joint gap after repetitive pitching

**Figure 2:**
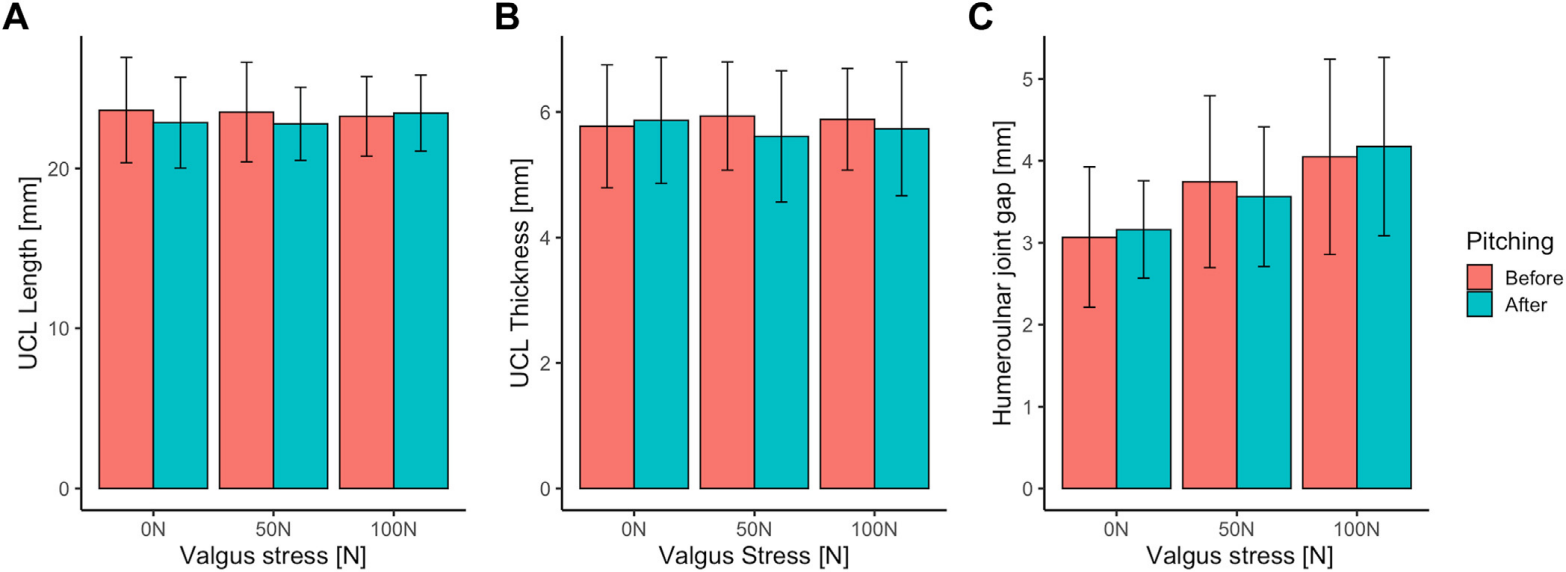
(A) and (B) show the UCL length and UCL thickness, respectively, for the different levels of applied static elbow valgus stress (0, 50, 100N) before and after repetitive pitching. (C) shows the humeroulnar joint gap before and after repetitive pitching. Error bars represent the standard deviations. *UCL*, ulnar collateral ligament.

Fig. 2*A* and *B* shows the mean UCL length and thickness, respectively, before and after repetitive pitching, for the different levels of elbow valgus stress without handgrip force. The mean UCL length varies from 22.85 (SD 2.84) mm before to 23.63 (SD 3.29) mm after repetitive pitching. The mean UCL thickness varies from 5.67 (SD .95) mm before to 5.93 (SD .86) mm after repetitive pitching. There was no significant main effect of repetitive pitching on UCL length or UCL thickness (Table III). There was also no significant main effect of elbow valgus stress on UCL length and UCL thickness, as well as no significant interaction with repetitive pitching.

**Table III:** Main and interaction effects of the two-way repeated measures ANOVA for repetitive pitching (before vs after repetitive pitching) and elbow valgus stress for UCL length, UCL thickness, and the humeroulnar joint gap without handgrip force.

	F-value (df)	*P* value	Effect size (η^2^)	Bonferroni		
				0N–50N	0N–100N	50N–100N
**Repetitive pitching**						
UCL length	2.06 (1, 14)	*P* = .17	.129			
UCL thickness	1.67 (1, 14)	*P* = .22	.107			
Humeroulnar joint gap	0.11 (1, 12)	*P* = .75	.009			
**Valgus stress**						
UCL length	0.12 (2, 28)	*P* = .89	.008			
UCL thickness	0.09 (2, 28)	*P* = .91	.007			
Humeroulnar joint gap	16.81 (2,24)	*P* < .001∗	.584	*P* = .011	*P =* .003	*P =* .008
**Repetitive pitching∗ valgus stress**						
UCL length	1.22 (2, 28)	*P =* .31	.080			
UCL thickness	1.77 (2, 28)	*P =* .19	.112			
Humeroulnar joint gap	3.30 (2, 24)	*P =* .06	.216			

*ANOVA*, analysis of variance; *UCL*, ulnar collateral ligament.

Fig. 2*C* shows the results of the humeroulnar joint gap of 13 participants, before and after repetitive pitching, without the handgrip force. There was no significant main effect of repetitive pitching on the humeroulnar joint gap (Table III).

### Effect of handgrip force before and after pitching on the humeroulnar joint gap

**Figure 3:**
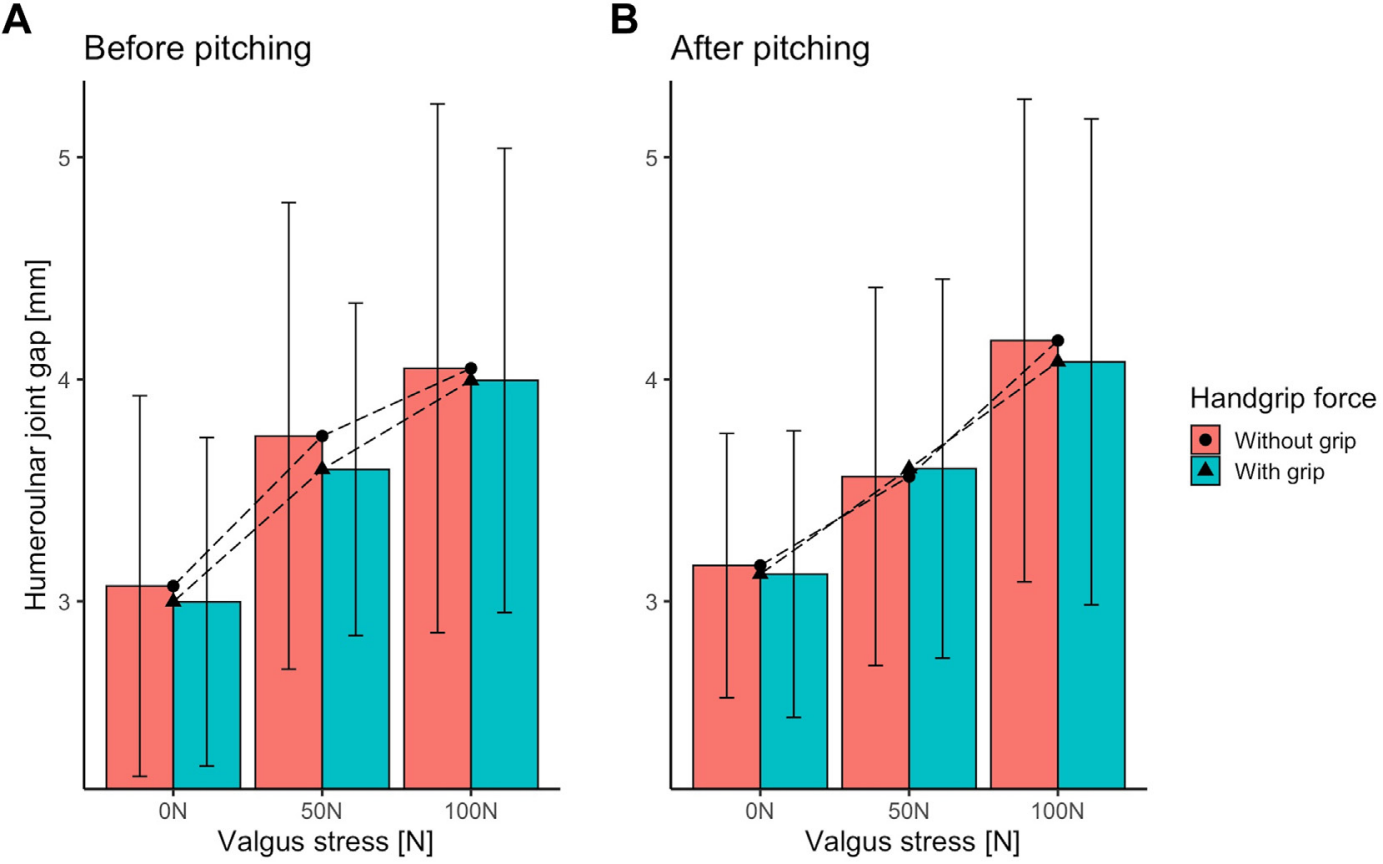
(A) shows the mean humeroulnar joint gap (in mm) for the different levels of elbow valgus stress (0, 50, 100 N) without and with handgrip force before repetitive pitching. (B) shows the humeral ulnar joint gap after pitching. To visualize interaction effects, the dashed line with the dots shows the condition without handgrip force and the dashed lines with the triangles show the condition with handgrip force. Be aware, because of visualizing the three-way repeated measures ANOVA, the y-axis does not start at zero. *ANOVA*, analysis of variance.

Data before and after repetitive pitching were analyzed to determine the effect of handgrip force on the humeroulnar joint gap at different levels of elbow valgus stress (Fig. 3). There was no significant three-way interaction between repetitive pitching (before vs after repetitive pitching), handgrip force, and elbow valgus stress with respect to the humeroulnar joint gap (F(2,24)=2.30, *P* = .122, η^2^ = 0.161).

## Discussion

The aims of the study were to investigate the effect of handgrip force and repetitive pitching on the humeroulnar joint gap size and UCL morphology and to investigate whether handgrip force differently affects the humeroulnar joint gap for different levels of elbow valgus stress after, and also compared to before, repetitive pitching in asymptomatic baseball pitchers. No significant effect of the exertion of handgrip force on the humeroulnar joint gap for different levels of elbow stress was observed. In addition, the UCL morphology and humeroulnar joint gap were also not significantly affected by repetitive pitching for the different levels of elbow stress. Finally, the nonsignificant three-way interaction indicated that the change in humeroulnar joint gap for the combinations of handgrip force and elbow valgus torque was not different after repetitive pitching compared to before the pitching session.

As expected, and in concordance with the literature,^16^ the humeroulnar joint gap increased significantly with an increase in elbow valgus stress imposed by the TELOS device. This proves that the medial side of the elbow joint is loaded as a consequence of applied valgus stress. Valgus stress is resisted by the UCL and counteracted by elbow muscles. Thus, an increase in valgus stress results in an increase in UCL load and elbow muscle load. A static mechanical calculation shows that the valgus stress of 50N and 100N is comparable with 12.5Nm and 25 Nm, respectively. Assuming a distance of 25 cm from the handle on the forearm to the applied valgus stress (Fig. 1). This is lower compared to the 50Nm peak external valgus torque under dynamic circumstances while pitching.^1^^,^^24^ Thus, the humeroulnar joint gap might be even larger during pitching.

The elbow muscles have the potential to shield the UCL from high valgus torques while pitching.^25^ We expected to find a decrease in the humeroulnar joint gap in pitchers in relation to handgrip force as other studies showed a decrease in the humeroulnar joint while maximal gripping in a general population of healthy males.^12^^,^^20^ In contrast, the present results showed that the humeroulnar joint gap was not significantly reduced with handgrip force, independent of the levels of valgus stress imposed on the elbows of the participants in this study. The participants in our study, however, did not perform maximal handgrip force but applied a handgrip force of 20% of the maximum. Tsubono et al (2022), published after our measurements, defined 50% maximal handgrip force as a cutoff point at which changes in the humeroulnar joint gap can be measured in a general male population 26. Lower values of handgrip force could explain why we did not find a significant effect on the size of the humeroulnar joint gap. The flexor-pronator muscles are active during pitching and have the potential to stress shield the UCL 24. However, the magnitude of the force exerted by the elbow muscles seems related to the humeroulnar joint gap and thus may affect the extent of stress shielding during pitching. Therefore, future studies should investigate how elbow muscle force is related to the humeroulnar joint gap and thus with the UCL loading during pitching.

In a single session, the results did not reveal a significant effect of repetitive pitching for the different levels of valgus stress on the humeroulnar joint gap and UCL thickness and length. We expected, based on the results of Hattori et al (2018), that the humeroulnar joint gap would increase after a session of repetitive pitching without considering the effect of handgrip force. Despite a comparable ball speed, the pitchers in our study were eight years older compared to the high school pitchers in the study of Hattori et al Younger and less experienced pitchers, who have therefore been less exposed to mechanical load at their elbows in the past, have a thinner UCL and a more lax humeroulnar joint gap compared to older and more experienced pitchers.^3^ Older pitchers are exposed more frequently to higher magnitudes of valgus torques while pitching during their lifetime. Adaptations increase the strength of the UCL and possibly the elbow muscles and thus elbow the shielding effect of elbow muscles during pitching. This might explain why the frequency of pitch number might have more impact on the humeroulnar joint gap in younger pitchers compared to older pitchers. Another explanation for not finding an effect on elbow response might be that the exposure was too low. The participants were instructed not to throw at least two days before the measurements. Pitching 100 balls during training or a game is within the general exposure of a non-fatigued pitcher. An increase in exposure by pitching more balls might have shown an effect on elbow response in adult pitchers.

After repetitive pitching, the shielding effect of the elbow muscles might be reduced as a result of muscle fatigue. The results of the present study did not show that for the humeroulnar joint gap, the interaction between handgrip force and valgus stress was different after repetitive pitching compared to before the pitching session. On the one hand, this could mean that the forearm muscles are able to help stabilize the elbow joint after a single session of repetitive pitching in the same way as before. High school pitchers have shown a decrease in maximal handgrip strength after repetitive pitching, but this reduction was not correlated with the humeroulnar joint gap quantified under gravity stress and without handgrip force.^13^ This indicates that repetitive pitching does not influence muscle force in relation to the humeroulnar joint gap. On the other hand, as explained above, we did not find a decrease in the humeroulnar joint gap with the applied 20% of the maximal handgrip force, whereas higher percentages of handgrip force decreased the humeroulnar joint gap.^23^ The effect of fatigued muscles might become detectable at a higher magnitude of handgrip force. Therefore, it should be investigated if elbow muscles after repetitive pitching are less capable of counteracting valgus stress at higher percentages of handgrip force.

That the results did not show a decrease or increase in the humeroulnar joint gap, respectively, while gripping or after repetitive pitching does not necessarily mean that the forearm muscles are not counteracting the external valgus torque during pitching. Because if the muscles are not counteracting the valgus torque during pitching, we might have seen an increased humeroulnar joint gap as a response to repetitive pitching. Observed differences in forearm muscle activation between baseball pitchers with and without elbow symptoms may support this explanation. Glousman et al (1992) found a decrease in activation in the forearm muscles (flexor carpi radialis and pronator teres) in symptomatic pitchers compared to asymptomatic pitchers during pitching, which could be associated with a higher UCL load in the symptomatic pitchers during pitching. However, within a single pitching session, we are not able to detect changes in the elbow responses in asymptomatic baseball pitchers.

It is clinically relevant to understand the elbow muscle stress shielding effect and how alterations in the humeroulnar joint gap and maladapting in UCL morphology are related to UCL injuries. Static ultrasound of the UCL morphology and humeroulnar joint gap showed changes in the response to elbow stress between in- and off-season 4. Therefore, changes in thickness and humeroulnar joint gap seem valuable while measuring over a longer period to quantify the response to elbow stress instead of a single session. In addition, changes in the humeroulnar joint gap with handgrip force with an elbow exposed to valgus stress might become detectable as a reduction of elbow muscle force due to fatigue over a longer period instead of a single session.

The humeroulnar joint gap, the UCL thickness, and length did not respond to elbow stress after repetitive pitching in this study. This does not mean that the UCL does not respond to pitching. In situ studies showed microdamage in ligaments under submaximal loading. Comparable microdamage in the UCL morphology cannot be detected with the measurement setup in this study. However, changes in the ligament morphology might become visible after a few hours or a few days as an inflammatory reaction might thicken the ligament.^15^ Only the elbow response was measured directly after pitching which is a limitation of this study. Another limitation of this study is that ultrasound imaging may seem an objective method to assess properties of anatomical structures, but it is associated with rater-dependent measurement error when taking the actual images and when analyzing the images for distances. Therefore, investigating differences within an individual becomes difficult because measurement errors might be larger than differences or changes that are clinically relevant. To limit the measurement error, the same static valgus stresses with the use of a TELOS device were applied, and bony landmarks were marked to locate the arm in the same position before and after pitching. While analyzing the data, our results showed moderate to good intrarater reliability for all three outcome variables, comparable with other studies.^4^,^12^

## Conclusion

The humeroulnar joint gap increases with increasing levels of static elbow valgus stress. Handgrip force, used as a proxy for the stabilizing effect of the flexor pronator mass muscle, did not affect these changes in the humeroulnar joint gap, and higher levels than 20% of the maximal handgrip force are likely needed to decrease the joint gap. In asymptomatic pitchers, repetitive pitching did not influence the humeroulnar joint gap, the UCL morphology, or the interaction between handgrip force and elbow valgus stress. To conclude, adult baseball pitchers do not respond to elbow stress after a single pitching session in the humeroulnar joint gap and UCL length and thickness. Clinically, it seems more relevant to quantify the elbow response over sessions and seasons while considering the elbow muscle forces.

## Disclaimers:

Funding: No funding was disclosed by the authors.

Conflicts of interest: The authors, their immediate families, and any research foundation with which they are affiliated have not received any financial payments or other benefits from any commercial entity related to the subject of this article.

## Data availability

The data underlying this study can be found here DOI: 10.4121/22140041
